# Addressing Climate Hazards and Resilience in the Greater Boston Inner Core: Semiautomated Content Analysis of Municipal Reports

**DOI:** 10.2196/100771

**Published:** 2026-11-12

**Authors:** Nina Cesare, Chad W Milando, Allison M James, Muskaan Khemani, Madeleine K Scammell, Beverly Ge, Mariangelí Echevarría-Ramos, Amruta Nori-Sarma, Jonathan I Levy

**Affiliations:** 1 Center for Health Data Science, School of Public Health, Boston University, 715 Albany Street, Boston, MA, 02118, United States, 1 617-358-2281; 2 Department of Environmental Health, School of Public Health, Boston University, Boston, MA, United States; 3 Mystic River Watershed Association, Arlington, MA, United States; 4 T.H. Chan School of Public Health, Harvard University, Boston, MA, United States

**Keywords:** climate resilience, community planning, hazard identification, outreach strategies, content analysis

## Abstract

Semiautomated web scraping and content analysis tools can be used to identify and summarize municipal climate reports, lending high-level insight into policy priorities across broad geographic and political units. A semiautomated content analysis of reports within the Greater Boston Inner Core uncovers homogeneity in climate priority and variation in community outreach efforts. Across the study area, reports highlighted floods and storms as focal hazards. Surveys and community meetings were common outreach tools, but municipalities varied in their approach. The analytic framework and tools used in this analysis may be of use to others seeking to improve existing environmental policy or coordinate policy decisions across communities.

## Introduction

The frequency and severity of extreme weather events is increasing, and municipalities respond with strategies to protect residents [1]. Although communities are unique, there may be overlaps in how neighboring communities perceive hazards and plan resilience strategies. Therefore, analysis of municipal reports can provide valuable insights for coordination.

Manual synthesis of municipal documents within a larger metropolitan area is time-consuming. Individual documents may differ in length, format, and online location. These challenges affect the consistency and completeness of identified hazards.

We present a semiautomated framework for acquiring relevant text and synthesizing climate hazards of concerns and outreach strategies used by communities within a given region. We employ API-based Google searches and content analysis, the process by which text-rich documents are converted into text that is computer-legible [2], to assess concerns raised by municipalities across the Greater Boston area [3]. This framework can be used to synthesize climate concerns across other major metropolitan areas.

## Methods

The 21 municipalities in the Greater Boston region (Figure 1) contain 1.6 million individuals across municipalities that range in population size (18,558 to 689,326) and median household income (US $68,331 to US $154,398). Common environmental hazards include urban heat islands and flooding [4]. We analyzed municipal reports addressing extreme weather resilience published between 2017 and 2024.

**Figure 1.**
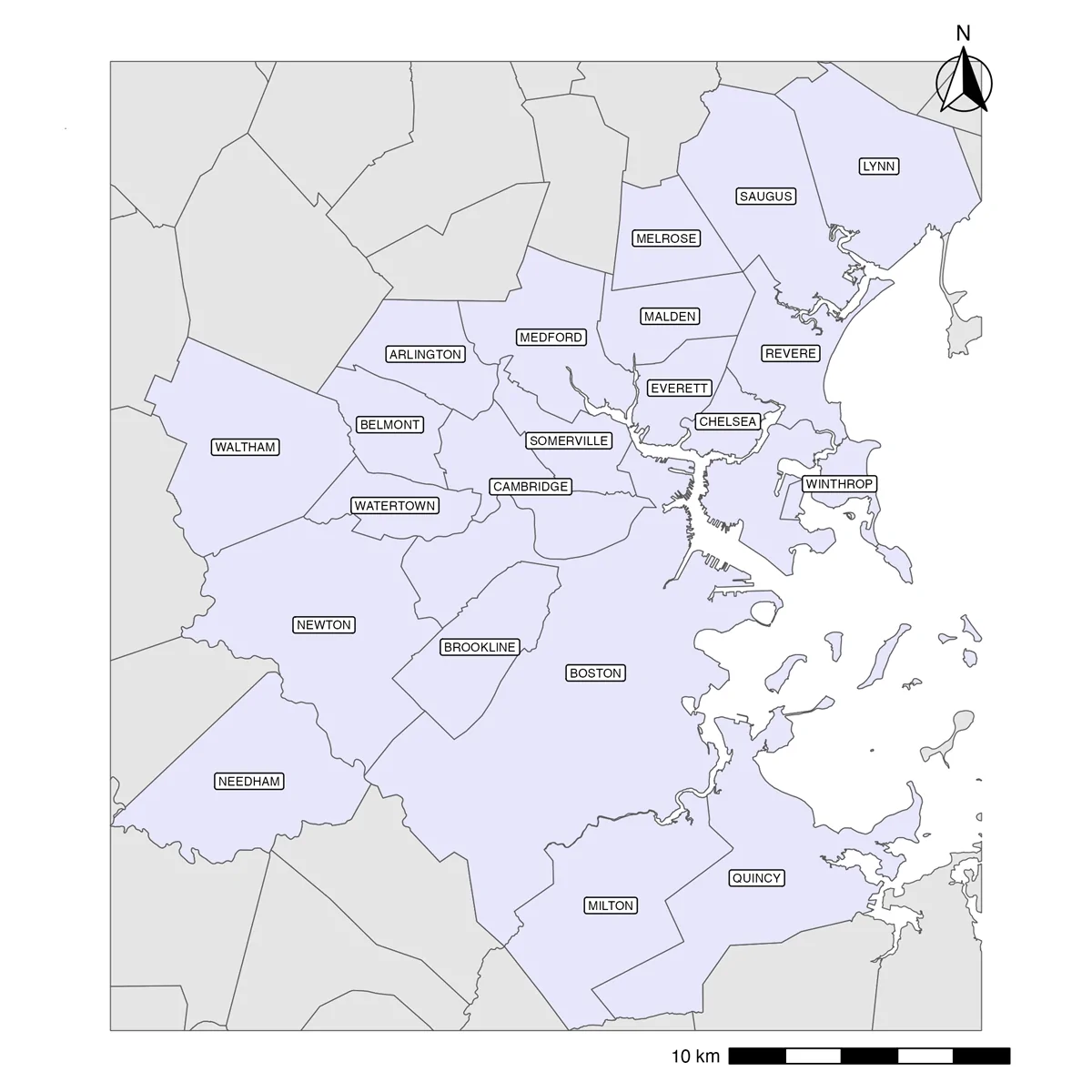
Map of the Greater Boston Inner Core.

We searched for climate resilience and hazard mitigation plans manually (Figure S1 in Multimedia Appendix 1) and via a Massachusetts government repository [5]. We then used Google’s Programmable Search Engine API [6], which provides search results without bias from user data [7]. We created queries combining community name and terms related to reporting, for example, “Boston” AND “climate” AND (“hazard” OR “resilience” OR “vulnerability”) AND “report.”

These queries resulted in 751 documents, which we filtered for relevance using Python (version 3.13.13) and R (version 4.5.1; R Foundation for Statistical Computing) scripts. We excluded reports where “Massachusetts” was not the most common US state mentioned, the most common town was not a Massachusetts town, and the words “community” or “climate” did not appear. We reviewed the remaining reports and kept 123. We took a convenience sample of 1 report per town and manually created a dictionary of key terms related to hazards of concern and strategies for community outreach (see Tables S1 and S2 in Multimedia Appendix 1). We then tokenized and normalized each PDF, calculated the frequency of each dictionary-based term, and calculated the total number of hazard or outreach terms within each document.

We then calculated metrics to compare the frequency of referenced hazards and outreach efforts across towns with varying quantities of climate documents and across documents with different lengths. We assessed the percentage of documents that contained a specific hazard or outreach term, calculated the proportion of terms related to each term, and calculated summary statistics of these proportions in the full region and within each municipality.

### Ethical Considerations

This study used publicly available reports to characterize priorities and trends across communities. Because these data were not gathered from individuals and involve no direct interaction with human participants, this study does not require human subjects research approval through the Boston University Institutional Review Board.

## Results

### Hazards of Concern

Hazard frequencies were similar across communities (Figure 2A). Flooding was mentioned proportionately the most within reports, accounting for a median of 44.7% (IQR 30.2%‐58.7%) of mentioned hazards per report. Next most common were storms (29.1%, IQR 20.5%‐38.4%), heat (4.5%, IQR 0.3%‐9.8%), and extreme precipitation (3%, IQR 1.2%‐5.8%).

**Figure 2.**
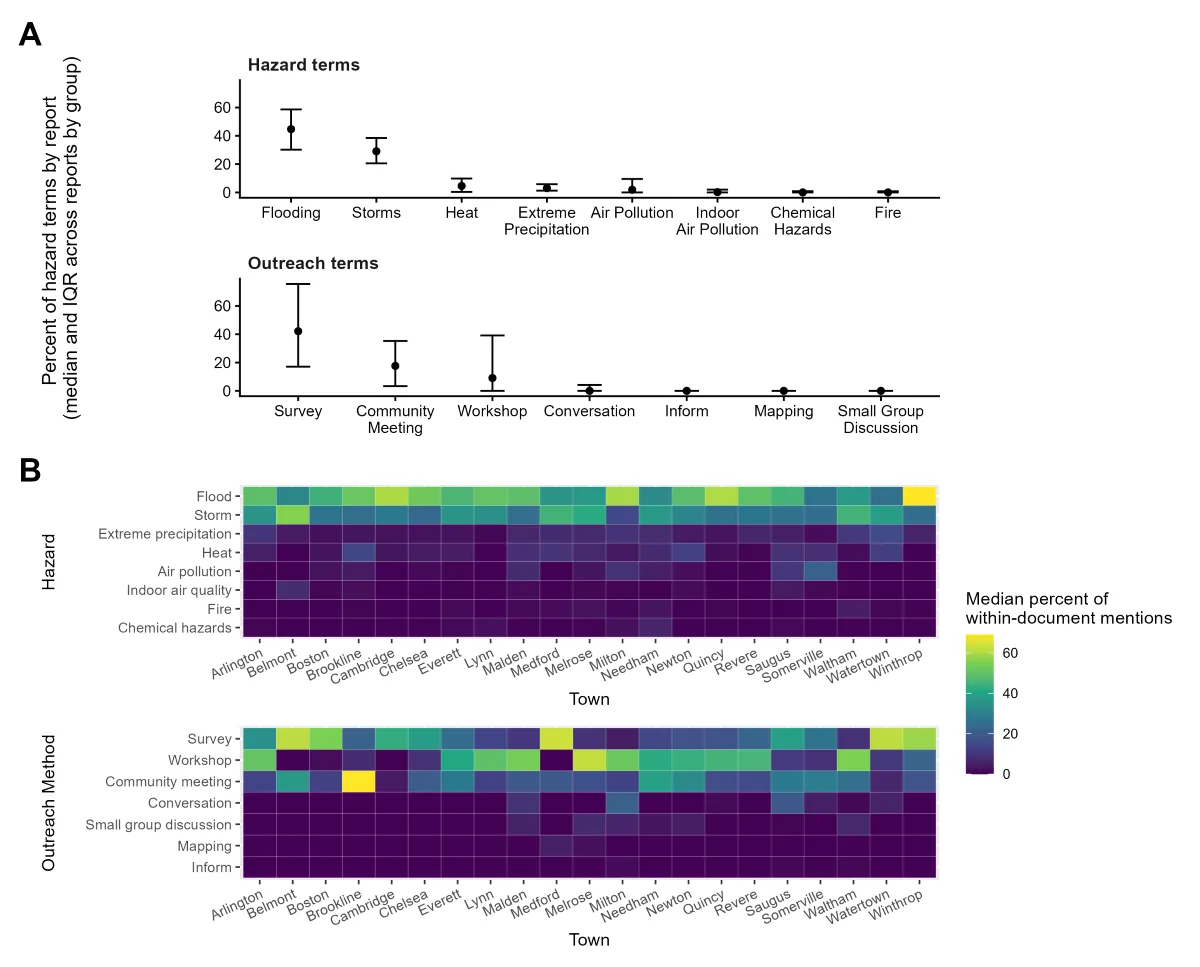
(A) Medians and IQRs of the per-report percentage of hazard terms represented by all reports in the Greater Boston Inner Core. (B) A heatmap representing the median per-report percentage of hazard and outreach terms across municipalities within the Greater Boston Inner Core.

Flooding and storms were also mentioned most often across reports, with each hazard appearing in 97.6% of reports. Extreme precipitation and heat were the third (86.2%) and fourth (79.7%) (see Table S3 in Multimedia Appendix 1). Air pollution, indoor air, chemical hazards, and fire were discussed less frequently.

### Reported Outreach Strategies

Surveys had the highest mentions by proportion within reports (Figure 2B), with a median proportion of 42.2% (IQR 17.1%‐75.5%) of all outreach terms. Community meetings amounted to 17.6% of per-report mentions (IQR 3.3%-32.3%), followed by workshops with 9.1% (IQR 0%-39.2%).

Surveys were also mentioned most often across reports, appearing in 94.3% of reports. Community meetings and workshops were also frequently mentioned, appearing in 78% and 65.9% of reports, respectively (see Table S3 in Multimedia Appendix 1).

Outreach strategies varied by community. For example, the IQR of per-report percent of survey terms was 17.1% to 75.5%, and for community meeting it was 3.3% to 35.3%.

## Discussion

### Principal Findings

Semiautomated methods captured and summarized municipal text addressing climate resilience in the Greater Boston Inner Core. Concerns related to climate hazards were consistent across the study area and predominantly cited flooding and storms, despite variation in population size, median income, and proximity to water. Though helpful for policy prioritization, homogeneous climate concerns may signify gaps in acknowledgment of other key hazards—for example, heat [8]. Reported outreach strategies were less consistent across communities, which may indicate opportunities for coordination across cities and towns.

### Comparison With Existing Studies

This study provides an approach to surveying past initiatives that is more scalable than a manual literature review and leverages content analysis as an effective means of analyzing municipal broadcasts. It builds upon Divay and Micheau’s [9] content analysis of citizen engagement and recognition by focusing on a policy area. It shares content analysis methodology with the assessment by Tang et al [10] of smart city archetypes and adds a semiautomated approach to document retrieval and analysis.

### Limitations

While our Google search methodology is generalizable, search criteria and restrictions may have impacted our sample. Additionally, our dictionary approach may miss references to hazards or outreach and does not account for hazard co-occurrence. While our qualitative evaluation of 21 initial climate resilience reports helped us anticipate document content, we may have missed terminology or conversations unique to specific municipalities. Additionally, the frequency of community outreach terms does not reflect the breadth, depth, or impact of outreach and may not represent on-the-ground engagement. Finally, we cannot infer the rationale behind highlighted hazards without additional policy context.

### Conclusions

As weather events become more extreme and communities work in parallel to develop action plans, community coalitions may use semiautomated techniques to identify and synthesize publicly available municipal reports related to climate hazards. With limited resources available for climate mitigation, pooling concerns could lead to shared strategies that benefit multiple communities. Additional research may explore how variation in policy discussion is tied to municipal characteristics such as population density, income, or proximity to natural environments.

## Supplementary material

10.2196/100771 Multimedia Appendix 1. 1Flowchart of document selection process; search terms used for hazards analysis; search terms used for outreach content analysis; and number of reports containing each hazard and outreach term.
