# Supplementary material for: Addressing Climate Hazards and Resilience in the Greater Boston Inner Core: Semiautomated Content Analysis of Municipal Reports
Source: JMIR Public Health Surveill. 2026 Nov 12;12:e100771. doi: 10.2196/100771 (PMC13759181; doi:10.2196/100771)
Supplement: Supplementary file 1 [file publichealth-v12-e100771-s001.docx]

# Multimedia Appendix 1: Supplementary Materials

**Figure S1.** Flowchart of document selection process. N manual = documents found by manual search; N state MVP = documents found on the state’s repository of municipal vulnerability preparedness (MVP) plans; N automated = documents found using the Programable Search Engine.

**Table S1**: Search terms used for hazards analysis

| **Hazard** | **Terms included** |
| --- | --- |
| Flood | "sea level rise", "riparian flooding", "tsunami", "flash flood", "inland flooding", "SLR", "rising sea", "coastal flooding", "flood", "inundation", "water levels", "storm surge", "high tide", "overflow", "floodwater", "flood plain", "flood risk", "flash flood", "riverine flooding", "precipitation-related flooding", "precipitation-based flooding" |
| Storm | "superstorm", "blizzard", "snowstorm", "winter storm", "tornado", "tropical storm", "Nor'easter", "coastal storm event", "hurricane", "wind", "storm", "cyclone", "typhoon", "gale", "storm damage", "severe weather", "thunderstorm", "tornado", "windstorm", "wind speed", "extreme weather" |
| Heat | “heat wave", "hot day", "high heat", "heat advisory", "heat stress", "high temperature", "heat stress", "thermal stress", "extreme heat", "hot weather", "temperature rise", "scorching temperatures", "heat-related illnesses", "vector borne diseases", "heat increase", "urban heat island", "UHI", "hot spot", "heat illness" |
| Air Pollution | "smog", "GHG", "air quality", "particulate matter", "PM2.5", "PM10", "NO2", "nitrogen dioxide", "sulfur dioxide", "SO2", "ozone", "VOCs", "volatile organic compounds", "airborne pollutants", "industrial pollution", "vehicle emissions", "exhaust fumes" |
| Indoor air quality | "mold", "asbestos", "radon", "carbon monoxide", "indoor pollution", "ventilation", "airborne particulates", "indoor allergens", "HVAC", "building sickness", "indoor VOCs", "formaldehyde" |
| Chemical hazards | "toxic waste", "hazardous materials", "chemical spill", "industrial waste", "contaminants", "biohazard", "radiation", "leakage", "pesticides", "herbicides", "heavy metals", "chemical exposure", "pollutants" |
| Extreme Precipitation | "torrential rain", "hail", "sleet", "drought", "heavy rainfall", "intense rainfall", "extreme rain events", "heavy downpour", "cloudburst", "deluge", "intense precipitation", "heavy snowfall", "record rainfall", "record snowfall", "flash flooding", "monsoon", "extreme weather conditions", "severe weather", "flood risk", "overwhelming rainfall", "high precipitation" |
| Fire | "wildfire", "brush fire", "forest fire", "fire hazard", "burning", "flame", "fire risk", "combustion", "flammable", "conflagration", "fire spread" |

Note: in this context we included “drought” in the extreme precipitation hazard category, given that this hazard is often discussed in the context of extreme flooding that occurs during drought conditions.

**Table S2**: Search terms used for outreach content analysis

| **Outreach Strategy** | **Terms included** |
| --- | --- |
| Workshop | "workshop", "workshops", "working group", "work group" |
| Mapping | "community mapping", "participatory mapping" |
| Conversation | "interview", "focus group" |
| Survey | "survey", "opinion poll", "poll", "questionnaire" |
| Community meeting | "town hall", "forum", "public hearing", "community meeting" |
| Small group discussion | "roundtable", "round table", "feedback session", "listening session" |
| Inform | "flyer", "pamphlet", "information session" |

**Table S3.** Number of reports containing each hazard and outreach term

| **Term grouping** | **Term** | **Number of reports that mention hazard (% of total)** |
| --- | --- | --- |
| Hazards | Flood | 120 (97.6%) |
|  | Storm | 120 (97.6%) |
|  | Extreme precipitation | 106 (86.2%) |
|  | Heat | 98 (79.7%) |
|  | Air pollution | 80 (65.0%) |
|  | Indoor air | 67 (54.5%) |
|  | Chemical hazards | 58 (47.2%) |
|  | Fire | 47 (38.2%) |
| Outreach | Workshop | 81 (65.9%) |
|  | Mapping workshop | 5 (4.1%) |
|  | Survey | 116 (94.3%) |
|  | Conversation | 44 (35.8%) |
|  | Community meeting | 96 (78.0%) |
|  | Small group discussion | 25 (20.3%) |
|  | Inform | 20 (16.3%) |
